# Expression profiling of ubiquitin-related genes in LKB1 mutant lung adenocarcinoma

**DOI:** 10.1038/s41598-018-31592-2

**Published:** 2018-09-05

**Authors:** Guanghui Wang, Fenglong Bie, Xiao Qu, Xudong Yang, Shaorui Liu, Yu Wang, Cuicui Huang, Kai Wang, Jiajun Du

**Affiliations:** 1 Institute of Oncology, Shandong Provincial Hospital Affiliated to Shandong University, 324 Jingwu Road, Jinan, 250021 P.R. China; 2 Department of Thoracic Surgery, Shandong Provincial Hospital Affiliated to Shandong University, 324 Jingwu Road, Jinan, 250021 P.R. China; 3 Department of Healthcare Respiratory, Shandong Provincial Hospital Affiliated to Shandong University, 324 Jingwu Road, Jinan, 250021 P.R. China

## Abstract

Liver kinase B1 (LKB1) is a tumor suppressor, and there is a very high proportion of LKB1 mutation in lung adenocarcinoma. The function of LKB1 is closely related to that of ubiquitin related genes. Our objective is to analyze the changes in ubiquitin-related genes in LKB1 mutant lung adenocarcinoma. We searched The Cancer Genome Atlas (TCGA) and obtained gene expression profiles from 230 lung adenocarcinoma patients, which were then analyzed using R software. Kaplan–Meier curves and Cox proportional hazards regression were applied to estimate survival. Real-time reverse transcription PCR was used to verify gene expression. Gene function was explored by gene set enrichment analysis. There were significantly expressed differences in the ubiquitin-related gene SH3RF1 between the LKB1 mutant and wild-type lung adenocarcinoma patients (p = 9.78013E-05). Patients with LKB1 mutation and high expression of SH3RF1 had a better prognosis than the low expression group (HR 0.356, 95% CI 0.136–0.929, p = 0.035). SH3RF1 can influence cell cycle, apoptosis, DNA replication and the p53 signaling pathway. SH3RF1 might have great clinical value act as a diagnostic biomarker and indicator to evaluate the prognosis of LKB1 mutant lung adenocarcinoma patients. This gene also can become a new treatment target for LKB1 mutant lung adenocarcinoma patients.

## Introduction

Lung cancer is one of the most common cancers and remains the leading cause of cancer-related death worldwide, leading to over a million deaths each year^1^. There are two types of lung cancer, small-cell lung cancer and non-small-cell lung cancer (NSCLC), and more than 80% of lung cancer patients are diagnosed with NSCLC^2^. Lung adenocarcinoma is the most commonly diagnosed histological subtype of NSCLC^3^. The LKB1 (liver kinase B1) mutation occurs in 19% of lung adenocarcinoma^4^. LKB1 mutation is commonly accompanied by changes in ubiquitination and deubiquitination genes^5^. A data analysis using The Cancer Genome Atlas (TCGA) revealed that there were many differentially expressed ubiquitination and deubiquitination-associated genes between the LKB1 mutant group and the wild-type group. Significant difference in expression was found in ubiquitin-related genes, including DCAF4, PML, TRAF3, PRKN, TRIM2, RAB40B, RNF187, SH3RF1, USP2 and etc.

The serine-threonine kinase 11 (STK11) gene, also called LKB1, is located on human chromosome 19p13.3, which contains 10 exons and codes for protein LKB1, which is composed of 433 amino acids^6,7^. LKB1 inactivation is one important cause of Peutz-Jeghers Syndrome, and LKB1 is also inactivated in approximately 25% of non-small cell lung cancers^8^. LKB1 is a protein kinase that can activate a family of 14 kinases related to the AMP-activated protein kinase (AMPK) pathway by direct phosphorylation^9^. LKB1 and AMPK are serine–threonine kinases implicated in key cellular pathways, including polarity establishment and energy sensing, respectively^10^. LKB1 is the second most commonly mutated tumor suppressor in sporadic human lung cancer (after TP53), especially in multiple subtypes of NSCLC^11^. There is evidence showing that the mutation rate of LKB1 is as high as 19% in adenocarcinoma, and the mutant ratio is tightly associated with the patients’ prognosis^4,8,12^. Our study focused on lung adenocarcinoma patients with LKB1 mutation and aimed to explore new diagnostic biomarkers to predict the prognosis for these patients.

Ubiquitin is a small protein that exists in all eukaryotes (in most eukaryotic cells), and the main function of ubiquitin is to mark proteins for degradation by hydrolysis^13^. Ubiquitination refers to the process that ubiquitin molecules undergo to classify intracellular proteins, choose target protein molecules, and specifically modify the target protein with the assistance of a series of special enzymes^14^. The conjugation of the 76-amino acid ubiquitin polypeptide requires the assistance of activating enzymes (E1s), conjugating enzymes (E2s), and ligase enzymes (E3s), resulting in combination between the C-terminus of ubiquitin and a specific lysine on the target protein^15,16^. Deubiquitination, the reverse of ubiquitination, refers to the process removing ubiquitin from modified proteins via deubiquitinating enzymes and is essential for the regulation of transcription, DNA repair, apoptosis, cell cycle progression, protein stability, and endocytosis^17^. The function of LKB1 is closely related to ubiquitin systems^18^, and therefore, we considered that ubiquitin system-related genes might have also changed when accompanied by the occurrence of LKB1 mutation.

We searched all the ubiquitination and deubiquitination genes and selected their expression information in patients based on TCGA. We found that there were significant expression differences in 116 genes. We performed survival analysis for each differentially expressed gene and found that there was a significant survival difference in the LKB1 mutant patient group for only 12 genes: USP2, CAP1, DCAF4, PML, PRKN, RAB40B, RNF168, RNF187, SH3RF1, TRAF3, TRIM2, and TRIML2. Then, we analyzed the gene’ expression, survival information, and population difference in the different groups and subsequently carried out real-time reverse transcription PCR (RT-PCR) to verify the expression of our target genes. After using gene set enrichment analysis (GSEA) to analyze the function of the target genes, we acquired one gene, SH3RF1.

SH3RF1 (SH3 domain containing RING finger 1), also known as POSH (plenty of SH3 domains), encodes a protein containing an N-terminus RING-finger, four SH3 domains, and a region implicated in the binding of the Rho GTPase Rac. Via the RING-finger, the encoded protein has been shown to function as an ubiquitin-protein ligase involved in protein sorting in the trans-Golgi network. SH3RF1 is a negative regulator of the death receptor and mediates apoptosis through the modulation of caspase-8 activity. The SH3RF1 gene may become a new diagnostic biomarker and treatment target in adenocarcinoma patients with the LKB1 mutation.

## Results

### Selecting target genes

**Figure 1:**
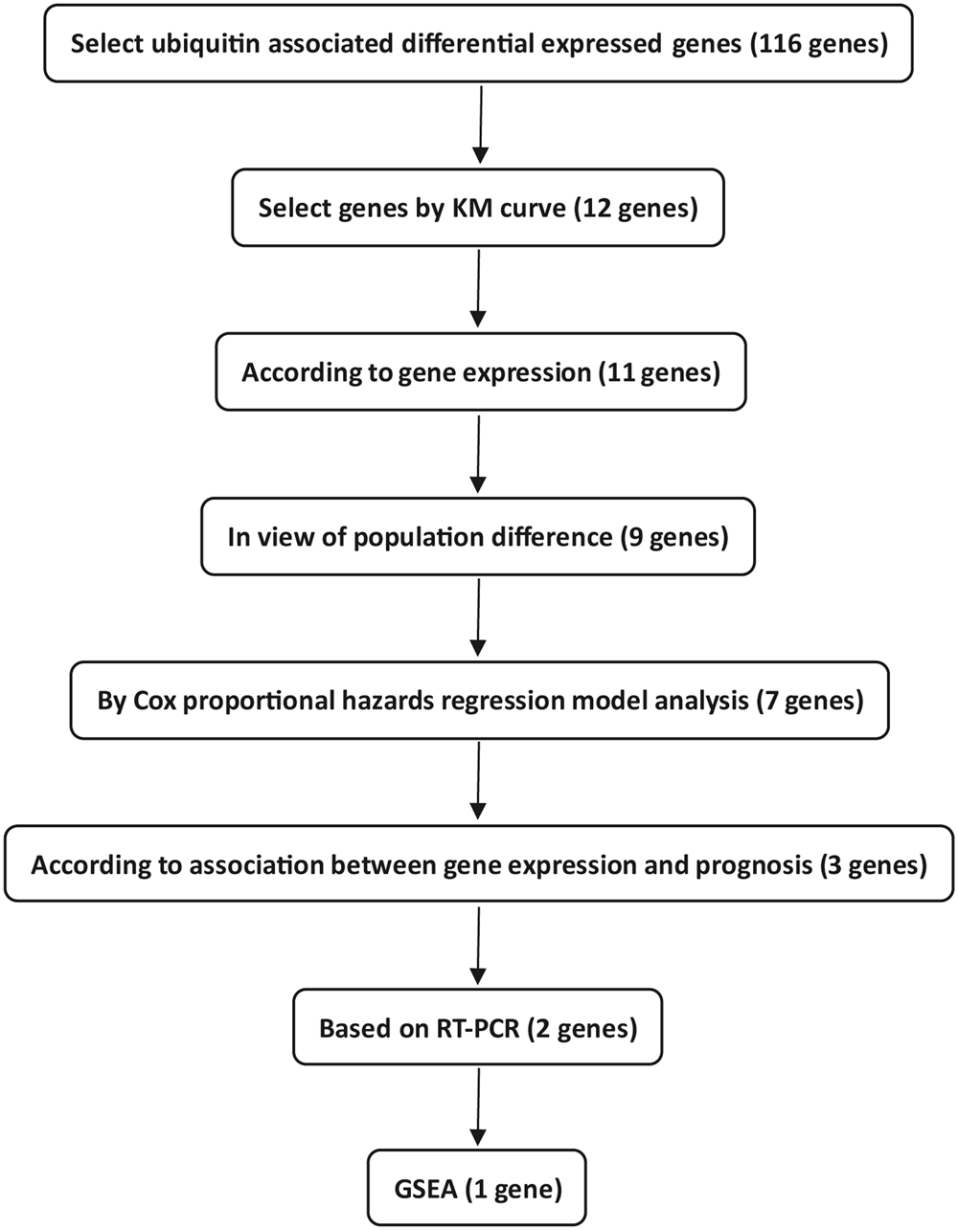
Flow sheet of the target genes screening process.

The flow sheet of the target gene screening process is showed in Fig. 1. We acquired 6429 differentially expressed genes from the R software running results. Then, we filtered out the deubiquitination and ubiquitination-related differentially expressed genes and acquired 116 genes (Supplemental Table 1). These differentially expressed deubiquitination and ubiquitination-related genes included 16 deubiquitinases, 7 E2 ubiquitin-conjugating enzymes, and 93 E3 ubiquitin-protein ligases. We selected genes that had significant differential survival in the LKB1 mutant patient group and had no significant differential survival in LKB1 wild-type patients, using p < 0.05 as the cutoff value. Only 12 genes, USP2, CAP1, DCAF4, PML, PRKN, RAB40B, RNF168, RNF187, SH3RF1, TRAF3, TRIM2, and TRIML2, were slected from 116 differential genes according to the Kaplan–Meier curve survival analysis. Then, we discard the TRIML2 gene from the set because its expression was too low for more than one half of patients. We removed the CAP1 and RNF168 genes due to their large population differences in the high expression and low expression groups of the LKB1 mutant group, and only 9 genes remained: USP2, DCAF4, PML, PRKN, RAB40B, RNF187, SH3RF1, TRAF3, and TRIM2.

We removed the USP2 and RNF187 genes because their Kaplan–Meier curve survival analysis results were not consistent with the Cox proportional hazards regression model analysis results. These two genes exhibited significant differential survival in the LKB1 mutant patient group, with no significant differential survival in LKB1 in the wild-type patient group, using p < 0.05 as the cutoff value, by Kaplan–Meier curve survival analysis. However, there were no significant differences in either the LKB1 mutant group or the LKB1 wild-type group in the Cox proportional hazards regression model analysis. The LKB1 mutation is a carcinogenic mutation and we thought that the changes in genes directly associated with LKB1 mutation should also be carcinogenic changes. However, for the DCAF4, PML, PRKN, and RAB40B genes, their expression changes in the LKB1 mutant group were favorable factors for prognosis in the Cox proportional hazards regression model analysis. Therefore, it was likely that these four genes might be indirectly regulated genes in LKB1 mutant patients, and we removed these four genes from the set. Thus, only 3 genes remained, SH3RF1, TRAF3, and TRIM2. We used RT-PCR to verify the expression of these three genes and found that TRIM2 and SH3RF1 had expressed differences. However, TRIM2 was removed by gene function analysis using GSEA and only SH3RF1 remained in the end.

### Expression profiling of mRNA

**Figure 2:**
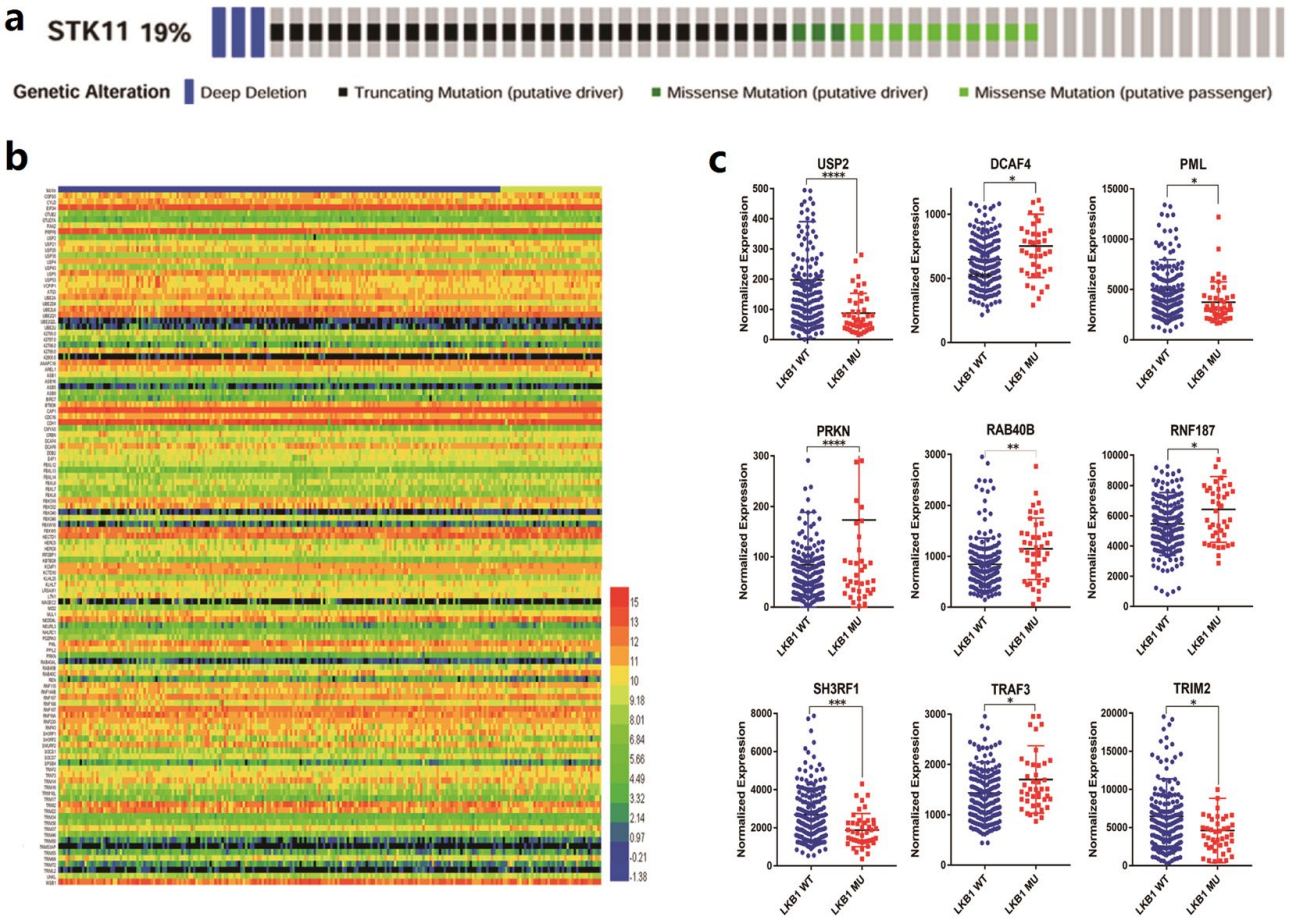
(**a**) STK11 gene alterations. Blue represents deep deletion, black represents truncating mutation (putative driver), black green represents missense mutation (putative driver) and green represents missense mutation (putative passenger). Genetic alterations were found in 43 of 230 lung adenocarcinoma patients (19%). The aberrant expression threshold was defined as z-score ± 2.0 from the TCGA RNA Seq V2 data. This OncoPrint was analysed by cBioPortal. (**b**) The heatmap of 116 ubiquitin related differential expression genes in 230 lung adenocarcinoma patients. The first row refers the LKB1 mutation information. Blue represents 187 LKB1 wild type patients and yellow represents 43 LKB1 mutation patients. **c**, the scatter plot of 9 ubiquitin related differential expression target genes. LKB1 WT represents the LKB1 wild type patients and LKB1 MU represents the LKB1 mutation patients.

We used the edgeR package by R software to analyze the TCGA database and selected 6429 differentially expressed genes from the R software running result. Then, we selected 116 deubiquitination- and ubiquitination-related genes from the 6429 differentially expressed genes. The differential expression information for these 116 genes is shown in Supplemental Table 1. Heml software was used to obtain the gene expression heatmap for these 116 genes (Fig. 2b). Rows refer to the gene names, and the different colors represent the different gene expression.

Because the differences in gene expression were not so significant, the heatmap did not directly show expression differences by color. Therefore, we selected 9 genes that were more representative: USP2, DCAF4, PML, PRKN, RAB40B, RNF187, SH3RF1, TRAF3 and TRIM2. Scatter plots were created (Fig. 2c) with GraphPad Prism 7 using normalized gene expression information for these 9 differentially expressed genes. The axis of the abscissa is labeled with the different groups, including the WT (LKB1 wild-type) group and MU (LKB1 mutant) group. The vertical axis is labeled with the normalized gene expression. All p values were obtained from a gene differential expression table called “edgerOut” that was created from the R software analysis.

### Prognosis analysis

**Figure 3:**
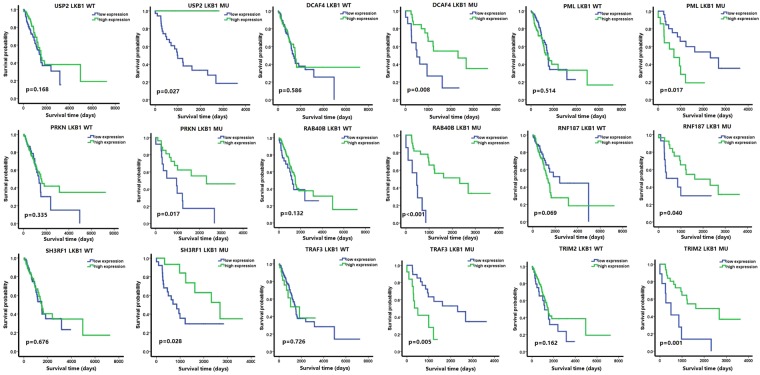
The Kaplan–Meier survival curve of 9 ubiquitin related differential expression target genes. WT represents the LKB1 wild type patients and MU represents the LKB1 mutation patients. All p values were two sides and less than 0.05 were considered significant.

ROC curve analysis was used to obtain appropriate cut-off values for 116 differential expression deubiquitination and ubiquitination-related genes. Then, we separated our patients into two subgroups consisting of high expression and low expression according to cutoff values in the LKB1 mutant and wild-type groups for different deubiquitination and ubiquitination-related genes. We selected 12 genes that exhibited significant differential survival in the LKB1 mutant patient group and had no significant differential survival in the LKB1 wild-type patient group; these genes were selected from 116 differentially expressed genes, using p < 0.05 as the cutoff value, and included USP2, CAP1, DCAF4, PML, PRKN, RAB40B, RNF168, RNF187, SH3RF1, TRAF3, TRIM2, and TRIML2. Due to expression that was too low or large population difference, TRIML2, CAP1, and RNF168 were all removed from the data set. The Kaplan–Meier survival curves for the other 9 genes are shown in Fig. 3. We also estimated patients’ survival prognosis risk by Cox proportional hazards regression model using HR > 1 or HR < 1 with p < 0.05 as the cutoff value. All analysis results are shown in Supplemental Table 2, and the analysis results for these 9 genes are shown in Table 1. Rows referred to gene names and columns were divided into LKB1 mutant and LKB1 wild-type groups. Every group included a HR (95% CI) (HR = hazard ratio, CI = confidence interval) and p value. We only selected genes in the LKB1 mutant group whose HR > 1 or HR < 1 with p < 0.05, and in the LKB1 wild-type group whose HR >  = 1 or HR < 1 with p > 0.05. We acquired 7 genes that met our standards: DCAF4, PML, PRKN, RAB40B, SH3RF1, TRAF3, and TRIM2. USP2 and RNF187 did not meet our standards.

**Table 1:** Analysis results of 9 genes by Cox proportional hazards regression model.

Gene	LKB1 mutation		LKB1 wild type	
	HR (95% CI)	p	HR (95% CI)	p
USP2	0.036 (0.000–3.990)	0.166	0.711 (0.437–1.157)	0.170
DCAF4	0.320 (0.133–0.769)	0.011	0.875 (0.540–1.416)	0.586
PML	2.826 (1.160–6.884)	0.022	1.172 (0.727–1.889)	0.515
PRKN	0.374 (0.162–0.866)	0.022	0.785 (0.479–1.286)	0.337
RAB40B	0.133 (0.046–0.386)	<0.001	0.658 (0.379–1.140)	0.135
RNF187	0.408 (0.169–0.986)	0.408	1.573 (0.962–2.572)	0.071
SH3RF1	0.356 (0.136–0.929)	0.035	0.902 (0.588–1.461)	0.676
TRAF3	3.485 (1.394–8.709)	0.008	1.134 (0.561–2.293)	0.727
TRIM2	0.253 (0.103–0.620)	0.003	0.675 (0.388–1.175)	0.165

All p values were two sides and less than 0.05 were considered significant. HR = hazard ratio, CI = confidence interval.

### Gene expression validated by western blot and RT-PCR

**Figure 4:**
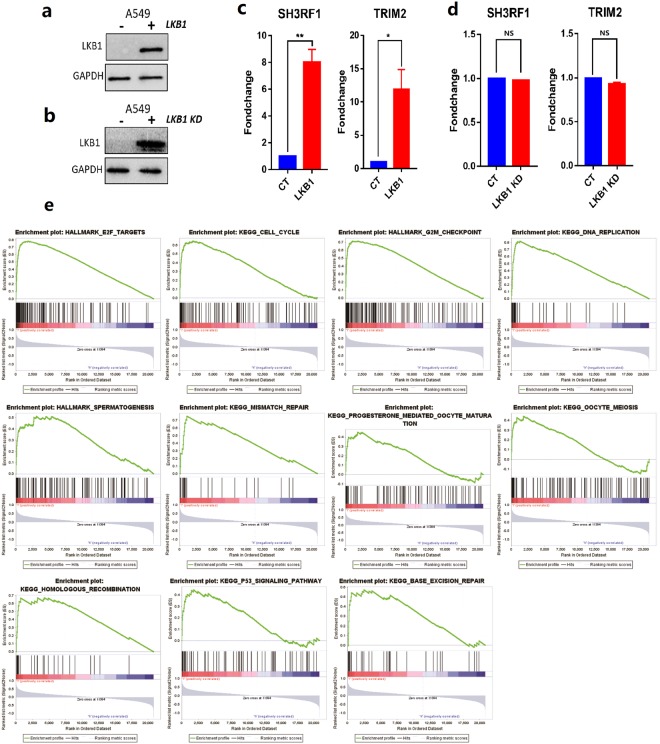
(**a**) western blot in A549 cell line. Cell lysates were collected, and anti-LKB1 antibody was used to detect LKB1 expression. GAPDH was used as the loading control. Negative sign refers to A549 stable transfection cell line transfected with pLenti-EF1a-mcherry-P2A-Puro-CMV-MCS-3Flag control plasmids. Positive sign represents A549 stable transfection cell line transfected with pLenti-EF1a-mcherry-P2A-Puro-CMV-stk11 plasmids. (**b**) negative sign refers to A549 transient transfection cell line transfected with PCDNA3.0 vector control plasmid. Positive sign represents A549 transient transfection cell line transfected with LKB1 (K78I) kinase-dead mutant plasmid. (**c**) Quantitative real-time PCR in A549 stable transfection cell line. TRIM2 and SH3RF1 primers were separately used to detect TRIM2 and SH3RF1 expression. CT refers to A549 stable cell line transfected with pLenti-EF1a-mcherry-P2A-Puro-CMV-MCS-3Flag. LKB1 refers to A549 stable cell line transfected with pLenti-EF1a-mcherry-P2A-Puro-CMV-stk11 plasmids. TRIM2, p = 0.0346. SH3RF1, p = 0.0089. All p values were two sides and less than 0.05 were considered significant. (**d**) Quantitative real-time PCR in A549 transient transfection cell line. TRIM2 and SH3RF1 primers were separately used to detect TRIM2 and SH3RF1 expression. CT refers to A549 transient transfection cell line transfected with PCDNA3.0 vector plasmid. LKB1 KD refers to A549 transient transfection cell line transfected with LKB1 (K78I) kinase-dead mutant plasmid. NS = not significant. (**e**) The GSEA results for SH3RF1 in LKB1 mutate NSCLC patients. Inclusion criteria: NOM p-val < 0.05 and FDR q-val < 0.25.

The LKB1 expression of established stable transfection A549 cells and transient transfection A549 cell lines was validated by Western blot, and the results are shown in Fig. 4. A549 cells are derived from a cell line with endogenous LKB1 deficiency. Consistent with this, LKB1 was undetectable in A549 cells by western blot. After we transfected the A549 cell line with control or LKB1 stable plasmids, we detected LKB1 in A549 cells, as shown in Fig. 4a. We also detected LKB1 in transient A549 cells transfected with LKB1 (K78I) kinase-dead mutant plasmid, as shown in Fig. 4b.

We verified expression using three target genes (SH3RF1, TRAF3, and TRIM2) via qRT-PCR, as shown in Fig. 4. We found that there were significant expression differences for genes SH3RF1 (p = 0.0089) and TRIM2 (p = 0.0346) between the control and LKB1 stable transfection A549 cell lines (Fig. 4c). However, there were no significant expression differences for genes SH3RF1 and TRIM2 between the control and LKB1 (K78I) kinase-dead mutant plasmid transient transfection A549 cells (Fig. 4d).

### Gene function enrichment analysis

We carried out gene function enrichment analysis for SH3RF1 and TRIM2 using GSEA software. For TRIM2, there was no significant result, and we subsequently removed this gene from the data set. Then, we used GSEA to analyze the function of SH3RF1 in NSCLC patients with the LKB1 mutation. We found 11 function-associated pathways that were significant in SH3RF1 low-expression lung adenocarcinoma patients with the LKB1 mutation (Table 2, Fig. 4e)

**Table 2:** GSEA results of SH3RF1 in LKB1 mutated NSCLC patients.

No	GS follow link to MSigDB	NES	NOM p-val	FDR q-val
1	HALLMARK_E2F_TARGETS	2.20	0.000	0.001
2	KEGG_CELL_CYCLE	2.15	0.000	0.001
3	HALLMARK_G2M_CHECKPOINT	2.15	0.000	0.001
4	KEGG_DNA_REPLICATION	2.08	0.000	0.003
5	HALLMARK_SPERMATOGENESIS	1.94	0.000	0.022
6	KEGG_MISMATCH_REPAIR	1.90	0.002	0.033
7	KEGG_PROGESTERONE_MEDIATED_OOCYTE_MATURATION	1.84	0.002	0.056
8	KEGG_OOCYTE_MEIOSIS	1.75	0.006	0.118
9	KEGG_HOMOLOGOUS_RECOMBINATION	1.72	0.016	0.136
10	KEGG_P53_SIGNALING_PATHWAY	1.71	0.015	0.129
11	KEGG_BASE_EXCISION_REPAIR	1.69	0.028	0.144

MSigDB = Molecular Signatures Database, NES = Normalized Enrichment Score, NOM p-val = Nominal p-value, FDR q-val = False Discovery Rate q-value. All p values were two sides and less than 0.05 were considered significant.

## Discussion

Although lung cancer is one of the most common cancers and remains the leading cause of cancer-related death worldwide, effective treatment modalities are still very inadequate and there is a lack of effective targeted therapy^19^. NSCLC accounts for approximately 85% of all lung cancer cases^20^. Lung adenocarcinoma is one of the most common lung cancers, with occurrence of up to 60% of all lung cancers^21^. Notably, the rate of the LKB1 mutation is very high in lung adenocarcinoma, and the LKB1 somatic mutation is detected in approximately 20% of lung adenocarcinomas^4^ (Fig. 2a). Many studies have reported that the function of LKB1 is closely related to ubiquitin and deubiquitin genes^5,22,23^. In our study, we examined ubiquitin-related differential expression genes with the goal of finding genes that would assist with the diagnosis of lung cancer and assessment of the lung cancer prognosis, and would also provide target treatment for lung cancer in the future.

There are many well-known mutations in lung cancer, including KRAS, p53, LKB1, and CDKN2A^24–29^. Our study focused on the LKB1 mutation in lung adenocarcinoma. LKB1, also called STK11, plays an important role in lung cancer, mainly through the AMPK pathway, to regulate cell proliferation, metabolism, apoptosis, cell polarity, and cell epithelial transformation^30–33^. Some studies have shown that many ubiquitin-related genes, including deubiquitination and ubiquitination-related genes, are closely associated with LKB1 function^34–37^. All of these studies showed that LKB1 may be regulated by ubiquitin-related enzymes. This greatly attracted our interest, and our study concentrated on elucidating ubiquitin-related gene changes in LKB1 mutant lung adenocarcinoma patients.

Some recent reports have noted associations with LKB1 by data mining using GEO (Gene Expression Omnibus) or TCGA. For instance, Chunxia Cao *et al*.^38^ concentrated on the function and regulated mechanism of cyclooxygenase-2 (COX-2) associated with LKB1. Nicolas Pécuchet *et al*.^39^ studied the prognosis value of the LKB1 mutation in in non-squamous NSCLC. Lu Chen *et al*.^40^ found that LKB1 might be a sensitive biomarker in clinical treatment based on NanoString. However, there have been few studies that systematically described ubiquitin-associated genes changes accompanied with the occurrence of LKB1 mutation in lung adenocarcinoma patients.

In our study, we concentrated on ubiquitin-associated gene changes in NSCLC patients with the LKB1 mutation. LKB1 mutation is a carcinogenic mutation, and therefore, it was likely that the expression changes in genes directly associated with LKB1 could result in a bad prognosis. However, we analyzed the 7 remaining genes (DCAF4, PML, PRKN, RAB40B, SH3RF1, TRAF3 and TRIM2) and found that 4 of them were inconsistent with this point of view. The expression changes of DCAF4, PML, PRKN, and RAB40B could lead to a better prognosis. After careful consideration, we recognized that these genes might be indirectly regulated by LKB1. Therefore, we moved our focus to the left genes, SH3RF1, TRAF3, and TRIM2, and applied RT-PCR to verify the gene expression for these genes. We found that there were significant expression differences for genes SH3RF1 and TRIM2 between the control and LKB1 stable transfection A549 cells. However, there were no significant expression differences for SH3RF1 and TRIM2 genes between the control and LKB1 (K78I) kinase-dead mutant plasmid transient transfection A549 cell lines, which indicated that the abnormally high expression of both SH3RF1 and TRIM2 genes were due to the function of the LKB1 gene, but not by the expression of ectopic proteins.

We explored gene function by enrichment analysis using GSEA software for the SH3RF1 and TRIM2 genes. We found that only SH3RF1-associated functional pathways exhibited significant differences in LKB1 mutant NSCLC patients. All of these differences might explain why SH3RF1 can affect the prognosis in LKB1 mutant lung adenocarcinoma patients. By analysis of the GSEA results, we concluded that SH3RF1 could influence apoptosis, cell cycle, DNA replication and repair, germ cell formation, and the p53 signaling pathway in LKB1 mutation lung adenocarcinoma patients. It was reported that SH3RF1 is closely related to cell cycle regulation and apoptosis in lung cancer^41^. Perry A *et al*.^42^ studied the function of SH3RF1 (SH3 domain containing RING finger 1), also known as POSH (plenty of SH3 domains), in apoptosis and demonstrated that SH3RF1 might act as an important mediator of death receptor mediated apoptosis. Traci R. *et al*.^43^ reported that SH3RF1 promoted apoptosis by acting as a scaffold. Philip Karuman *et al*. investigated the mechanism and function of LKB1, and demonstrated that LKB1 was physically associated with p53 and regulated specific p53-dependent apoptosis pathways^44^. JH Lee *et al*. showed that LKB1 negatively regulated organ growth by caspase-dependent apoptosis in Drosophila^45^. Ping Song *et al*. studied the causal role of oxidative stress in vascular injury in diabetes mellitus and concluded that hyperglycemia triggered apoptosis by inhibiting Akt signaling via LKB1-dependent PTEN activation^46^. All of these studies showed that LKB1 is closely related to apoptosis and induces apoptosis to some extent.

As mentioned earlier, SH3RF1 is a pro-apoptotic gene, while LKB1 is closely related to apoptosis. Therefore, we linked these two genes together and explored their relationship by examining the TCGA data and found that SH3RF1 is highly expressed in the LKB1 mutation group (as shown in Fig. 2c). Then, we verified the expression in A549 cells by qRT-PCR and found the same phenomenon (as shown in Fig. 4b). These data show that SH3RF1 is closely related to LKB1, and LKB1 may regulate some cellular biological behaviors (such as apoptosis) by regulating the expression of SH3RF1. Therefore, we can designate SH3RF1 as a new diagnostic biomarker and indicator that can be used to evaluate the survival and prognosis of LKB1 mutant lung adenocarcinoma patients. We can also consider SH3RF1 as a new therapeutic target that can be researched to discover new treatment methods for lung adenocarcinoma patients with the LKB1 mutation.

As far as we know, our study is the first to examine ubiquitin-associated genes in the LKB1 mutant and wild-type groups. Although we used TCGA database, which is a very authoritative database, there were problems in our study. Firstly, although our samples included 230 patients, they were small and we could not guarantee the accuracy of samples. Secondly, we only used TCGA database in our results. Although we also used the GEO database to verify our results, there was a very large difference in the number of the samples and results in different GEO databases. Therefore, we gave up analysis using GEO databases in the end. Additionally, although we used differential expression genes accordingly to adjust the p value, our selected genes did not have very large differences in gene expression levels because we abandoned the font change value as a screening criterion. Last but not least, although we carried out RT-PCR in our study, we lacked some other basic experiments to support our results.

Overall, we selected aberrantly expressed genes to estimate their prognosis value in lung adenocarcinoma patients with the LKB1 mutation. We found that there was great clinical value in the SH3RF1 gene and that it could act as a new diagnostic biomarker and indicator to evaluate survival and prognosis of LKB1 mutant lung adenocarcinoma patients. SH3RF1 can become a new treatment target and help us find new treatment methods for LKB1 mutant lung adenocarcinoma patients. However, additional experiments must be performed that will more deeply explore the underlying mechanism of SH3RF1 in LKB1 mutant lung adenocarcinoma.

## Patients and Methods

### Database source

TCGA is a public database (http://cancergenome.nih.gov/) that includes 29 cancer types, along with related gene expression and clinical information. The cBioPortal (http://cBioPortal.org) is a web-based public tool based on TCGA, where we acquired the LKB1 mutation information^47,48^. In this study, we used the TCGA database and cBioPortal tool to acquire mRNA expression data and survival information of lung adenocarcinoma patients. We downloaded our 230 lung adenocarcinoma patients from the Genomic Data Commons (GDC) of TCGA (https://portal.gdc.cancer.gov/) and searched for LKB1 gene mutantion information in the cBioPortal website. Then we separated these 230 lung adenocarcinoma patients into LKB1 mutant and LKB1 wild two groups.

### Construction of the LKB1 cell line

The adenocarcinoma cell line A549 was purchased from the American Type Culture Collection. Cells were cultured in Roswell Park Memorial Institute 1640 (RPMI 1640) medium purchased from Hyclone and supplemented with 10% fetal bovine serum; cells were grown at 37 °C in a humidified atmosphere with 5% CO_2_. Mouse monoclonal antibodies against LKB1 (sc-32245) and glyceraldehyde-3-phosphate dehydrogenase (GAPDH, sc-166545) were purchased from Santa Cruz Biotechnology. A549 cells, a cell line with endogenous LKB1 deficiency, were transfected with pLenti-EF1a-mcherry-P2A-Puro-CMV-MCS-3Flag (control) or pLenti-EF1a-mcherry-P2A-Puro-CMV-stk11 stable plasmids. The cells were then subjected to puromycin selection (4 ng/μl) for 2 weeks, after which we collected puromycin-resistant stable clones. The expression of LKB1 in established stable transfected A549 cells was validated by Western blot. We also transfected the LKB1 (K78I) kinase-dead mutant plasmid into the A549 cell line by means of transient transfection and used the A549 cell line transfected with PCDNA3.0 vector plasmid as a control. We verified the expression of LKB1 by western blot.

### Western blot

Cells were lysed in lysis buffer, and the protein concentration was determined by the bicinchoninic acid (BCA) protein assay. Equal amounts of protein from each cell lysate were subjected to sodium dodecyl sulfate-polyacrylamide gel electrophoresis (SDS-PAGE) and transferred onto polyvinylidene difluoride (PVDF) membranes. The membranes were blocked in 5% bovine serum albumin (BSA) for 1 hour at room temperature and then probed with primary antibodies against LKB1 (dilution 1:2000), GAPDH (dilution 1:1500) in Tris-buffered saline containing 0.2% Tween 20 and 5% fat-free dry milk overnight at 4 °C. After washing, the membrane was incubated with horseradish peroxidase-conjugated secondary antibodies (dilution 1:10000) for 1 hour at room temperature. Specific proteins were visualized with enhanced chemiluminescence detection reagent according to the manufacturer’s instructions.

### Real time RT-PCR

The expression of mRNA was examined by RT-PCR with the LightCycler/LightCycler 480 Real-time PCR System, using SYBR Premix DimerEraser (Takara, Japan) reagent in a 20 ml reaction volume. The primers for RT–PCR were designed by Primer3. The primer sequence is listed in Supplemental Table 3. Each sample was amplified in triplicate and normalized to 18S rRNA expression. The results were evaluated by the comparative threshold cycle value method (2^−ΔΔCt^) for relative quantification of gene expression.

### Expression profiling of mRNA

We used R software to perform gene differential expression analysis of TCGA database. We acquired the gene differential expression information using R software with the edgeR package. We selected 6429 differentially expressed genes from the R software running result, and used p < 0.05 as the cutoff value.

After we obtained our differentially expressed genes, we selected target genes consisting of deubiquitination and ubiquitination-related differentially expressed genes. Our 90 deubiquitination target genes were from Researchgate (https://www.researchgate.net) and included 5 families: USPs (57 genes), UCHs (4 genes), OTUs (14 genes), MJDs (4 genes), and JAMMs (11 genes). Our 490 ubiquitination target genes were from UniProt (http://www.uniprot.org/) and included E2 ubiquitin-conjugating enzyme, E3 ubiquitin-protein ligase, and other ubiquitin-associated genes. We ultimately selected 116 deubiquitination genes and ubiquitination-related differential expression genes.

After we completed the gene differential expression analysis, we also acquired a normalized gene expression text file in the running results. We selected information regarding our 116 differential genes from the normalized gene expression. We used Heml software to create a gene expression heatmap. Then, we used GraphPad Prism 7 software to create scatter plots using this normalized gene expression information.

### Statistical analysis

We performed a survival receiver operating characteristic (ROC) curve analysis to obtatin appropriate cut-off values for the 116 differentially expressed deubiquitination and ubiquitination-related genes using IBM SPSS Statistics 20. The most optimal cutoff value of the prognostic score was determined in the ROC curve analysis by patient survival time and gene expression. Then, we divided patients into high-expression and low-expression two subgroups according to cutoff values in the LKB1 mutant and wild-type groups for different deubiquitination and ubiquitination-related genes. We carried out an overall survival (OS) analysis for the LKB1 mutant group and wild-type group using the Kaplan–Meier and log-rank method by IBM SPSS Statistics 20. We selected genes that had significant differential survival in the LKB1 mutant patients group and had no significant differential survival in the LKB1 wild-type patient group, using p < 0.05 as the cutoff value.

We also assessed the patients’ survival prognosis risk using the Cox proportional hazards regression model and designating HR > 1 or HR < 1 with p < 0.05 as the cutoff value. Using the same procedure as that for selecting genes for the Kaplan–Meier survival curve, we selected genes in the LKB1 mutant patient group whose HR > 1 or HR < 1 with p < 0.05, and in the LKB1 wild-type patient group whose HR > = 1 or HR < 1 with p > 0.05.

### Gene function enrichment analysis

We used GSEA (gene set enrichment analysis) to explore the function of selected genes. GSEA is software that can provide scores based on gene expression and acquired pathways associated with gene function. In our study, we used two gene set databases, h.all.v6.0.symbols.gmt and c2.cp.kegg.v6.0.symbols.gmt, to analyze our target genes with GSEA 3.0. After we divided LKB1 mutant NSCLC patients into two groups according to the expression of our target genes, we separately analyzed the function of our two target genes, TRIM2 and SH3RF1.

### Novelty and Impact Statements

This is the first research study to explore ubiquitin-associated genes in LKB1 mutant and wild-type groups. We found that SH3RF1, an E3 ubiquitin-protein ligase, might be directly up-regulated by LKB1. SH3RF1 has great clinical potential to act as a new diagnostic biomarker and indicator for evaluation of the survival and prognosis in lung adenocarcinoma patients with LKB1 mutation, and it is also a potential therapeutic target.

## Electronic supplementary material

Supplementary Material

## Data Availability

All our data is available in the TCGA public database (http://cancergenome.nih.gov/).
