# Supplementary material for: Expression profiling of ubiquitin-related genes in LKB1 mutant lung adenocarcinoma
Source: Sci Rep. 2018 Sep 5;8:13221. doi: 10.1038/s41598-018-31592-2 (PMC6125361; doi:10.1038/s41598-018-31592-2)
Supplement: Supplementary file 1 — Supplementary Material [file 41598_2018_31592_MOESM1_ESM.pdf]

Expression profiling of ubiquitin-related genes in LKB1 mutant lung adenocarcinoma

Guanghui Wang, MD[1,2 +], Fenglong Bie, MD[1, +], Xiao Qu, MD[1], Xudong Yang, MD[1], Shaorui Liu, MD[1], Yu Wang, MD[1], Cuicui Huang, MD[1], Kai Wang, MD[1,3], Jiajun Du, MD, PhD[1,2,] *

1 Institute of Oncology, Shandong Provincial Hospital Affiliated to Shandong University, 324 Jingwu Road, Jinan, 250021 P.R. China

2 Department of Thoracic Surgery, Shandong Provincial Hospital Affiliated to Shandong University, 324 Jingwu Road, Jinan, 250021 P.R. China

3 Department of Healthcare Respiratory, Shandong Provincial Hospital Affiliated to Shandong University, 324 Jingwu Road, Jinan, 250021 P.R. China

* Corresponding author: Jiajun Du, Department of Thoracic Surgery and Institute of Oncology, Shandong Provincial Hospital Affiliated to Shandong University, 324 Jingwu Road, Jinan, 250021 P.R. China.

Tel: +86-0531-6877-7100; Fax: +86-0531-6877-7100; E-mail: dujiajun@sdu.edu.cn

[+] Guanghui Wang and Fenglong Bie contributed equally to this work.

Supplemental Fig. 1

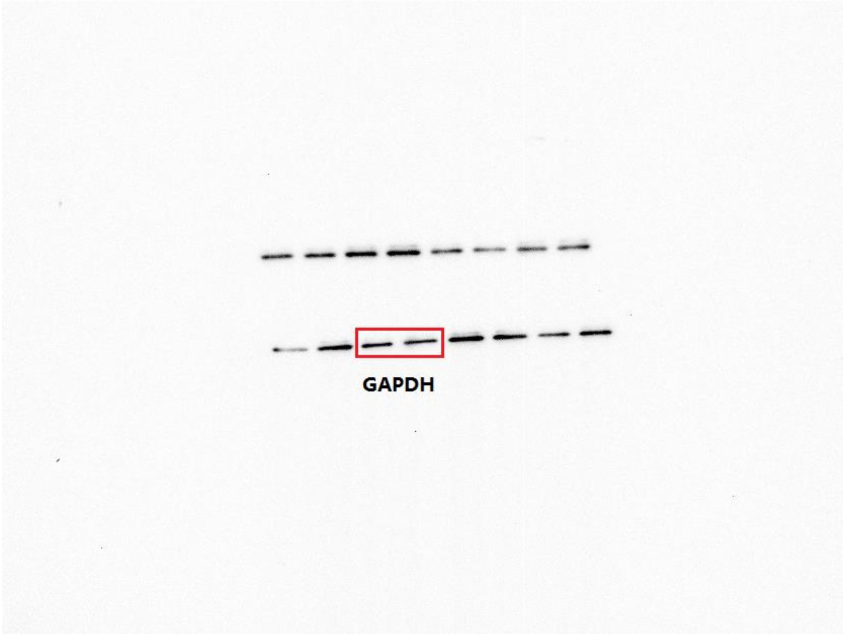

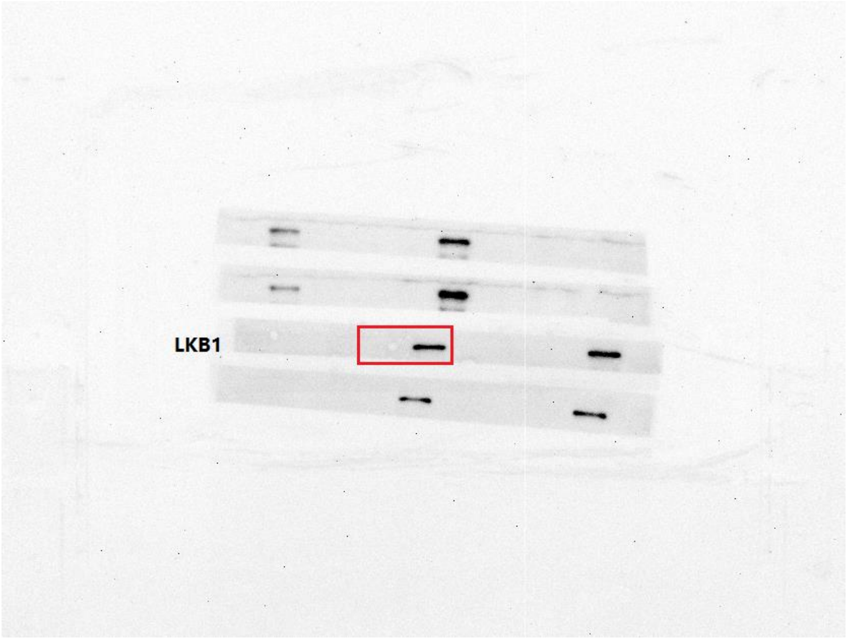

Supplemental Fig. 2

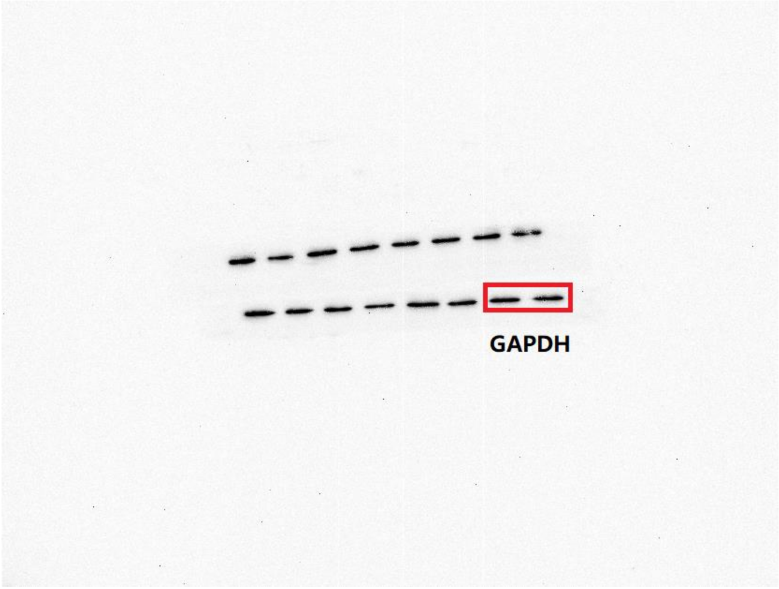

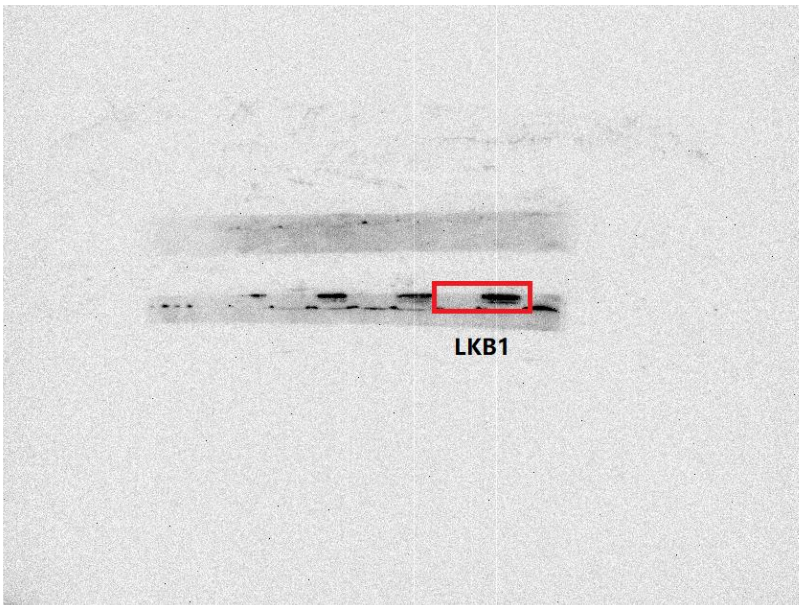

Supplemental Table 1 Differential expression analysis results of 116 genes

gene	logFC	logCPM	PValue	FDR
DUBs (16)				
USP2	-1.158720037	2.065576666	2.18683E-07	7.4524E-06
USP21	0.427189235	5.108062173	1.25617E-06	2.9455E-05
USP35	0.416113377	3.123663736	4.86988E-05	0.00055109
COPS5	0.428096483	5.995107462	6.15247E-05	0.000664454
EIF3H	0.400604982	8.189551449	0.000130085	0.001201695
OTUD7A	0.684181136	0.59099015	0.000395636	0.0029017
PRPF8	0.311839255	8.350918058	0.001130867	0.006601322
OTUB2	0.483779328	2.177332346	0.001274867	0.007235018
USP25	-0.321439555	5.846491543	0.001598806	0.008658087
USP43	0.376008442	3.465864668	0.00207552	0.010662837
PAN2	0.341202175	4.964856962	0.002785957	0.013413702
VCPIP1	0.272883662	5.472476294	0.007786617	0.02973825
USP4	-0.191501335	6.011143081	0.008099947	0.030603721
CYLD	-0.267383056	5.864354839	0.008723831	0.032396579
USP53	-0.332538142	5.630479753	0.010653696	0.037827284
USP5	0.262812926	6.663474769	0.013396085	0.044887879
E2s (7)				
UBE2Q1	0.346263236	6.944925473	1.08191E-05	0.000167967
UBE2U	-2.158481513	-2.122233538	0.000246641	0.001996721
UBE2D4	-0.27055581	4.737050829	0.00525683	0.022023685
ATG3	-0.193832468	5.625771914	0.008957385	0.033063406
UBE2Q2L	0.769975384	-3.729235461	0.009151029	0.033664794
UBE2A	-0.244550177	6.438526717	0.010837166	0.038323958
UBE2L6	-0.343194188	6.813877563	0.010945067	0.038639908
E3s (93)				
MARCH-4	3.312247816	1.382486384	1.18072E-23	2.45849E-20
TRAF2	0.952463913	4.972624602	3.40707E-17	2.14976E-14
ASB1	0.831666565	4.778358901	8.14141E-12	1.52721E-09
TRIML2	2.701128092	-3.527750265	2.18104E-11	3.29085E-09
REN	-2.047625305	-0.521536427	4.42082E-11	6.05594E-09
FBXW5	0.720922288	6.984129756	3.91607E-09	2.77349E-07
TRIM53AP	2.883555536	-3.401874244	1.09763E-08	6.45617E-07
MARCH-1	0.699590004	4.632309473	7.89029E-08	3.30567E-06
TRIM37	0.561186182	5.274082346	8.14092E-08	3.38938E-06
NEDD4L	0.779614603	7.106904784	1.00632E-07	3.961E-06
PRKN	1.066320589	1.269640172	1.16917E-07	4.49989E-06
SPSB4	-1.504638494	-0.80457923	3.39269E-07	1.05862E-05

RAB40C	0.63184687	5.873011189	4.4149E-07	1.31206E-05
HERC5	-0.840840083	3.842917404	4.45284E-07	1.317E-05
TRIM55	-1.868707168	0.251059017	5.98278E-07	1.63672E-05
MAGEC2	-5.071114353	0.835713865	1.02225E-06	2.51005E-05
FBXL13	-1.277733915	1.026857795	1.0809E-06	2.61703E-05
SH3RF2	-1.447220325	4.077612441	1.48065E-06	3.39165E-05
FBXO40	-2.716144769	-2.325687697	5.61364E-06	9.76164E-05
TRIM22	-0.664002312	6.467452333	9.69167E-06	0.000153111
SMURF2	-0.564978633	6.218369879	1.57711E-05	0.000229487
SOCS7	0.538804827	4.887421028	1.59919E-05	0.000231399
MARCH-5	0.308813344	5.792400848	1.78542E-05	0.00025153
FBXO46	0.395152924	4.437956747	1.81819E-05	0.000254424
TRIM16L	1.009833793	4.062618383	1.82129E-05	0.000254687
KBTBD8	-0.728949287	1.952866746	2.20449E-05	0.000297292
BTBD6	0.414268376	6.156361655	3.75561E-05	0.000454383
HERC6	-0.568346697	4.917674029	5.99569E-05	0.000650898
FBXL6	0.550015169	4.543208407	9.76548E-05	0.000956879
SH3RF1	-0.490871345	5.894166644	9.78013E-05	0.000957864
DCAF8	0.344938479	6.1454494	0.00012886	0.001194192
FBXO32	-0.72240976	6.046099951	0.000131398	0.001210453
MARCH-3	-0.635382493	2.621900021	0.000181018	0.001560729
KCTD10	-0.246461385	6.279711946	0.000271139	0.002150684
KLHL7	-0.415790568	5.250012644	0.000286596	0.00224342
ANAPC16	0.340922454	7.011708593	0.000288517	0.002251685
MUL1	-0.34772037	5.360684598	0.000350317	0.002629404
IRF2BP1	0.412189036	4.804084572	0.000387894	0.002857556
ASB16	0.556097551	0.597158231	0.000462576	0.003271657
RAB40B	0.464215741	4.412059855	0.000484287	0.003395226
TRIM46	0.636140161	2.284555531	0.000500752	0.003489513
LTN1	-0.365181491	5.330142855	0.00063017	0.004174204
ASB5	-1.368742886	-3.026248141	0.000661496	0.004336799
PPIL2	0.288988803	5.721448544	0.000815801	0.00511491
TRIM14	-0.393766229	5.86465625	0.000986395	0.005936048
BIRC7	-0.945878503	0.871130984	0.001080328	0.006352608
CMYA5	-0.681324304	2.36688136	0.00121711	0.006993009
MID2	-0.418259615	3.035662894	0.001396036	0.007774339
TRIM72	1.308039411	1.326690169	0.001494532	0.008219532
ASB9	0.599697535	3.114724758	0.0017357	0.009252231
RNF167	0.287293677	6.605974202	0.001801885	0.009527388
FBXL8	0.428731339	2.895669224	0.001804338	0.009537934

CAP1	-0.262254989	8.790707213	0.001975235	0.010256444
TRIM50	-1.145543404	-1.701726713	0.002360588	0.011835341
PML	-0.399756357	6.803720671	0.002496142	0.012360868
RNF144B	-0.421009767	5.290368872	0.002562682	0.012611714
LRSAM1	0.276304319	4.586088843	0.002715219	0.013184768
RAB40AL	-1.162705144	-2.940321496	0.002921487	0.013961718
TRIM17	0.810464445	2.277990493	0.002971582	0.014149162
CDH1	-0.418494435	9.032953175	0.003164351	0.01487988
AREL1	0.262800901	5.808597213	0.003179464	0.014923986
TRIM34	-0.457173777	0.130377646	0.003309981	0.015411542
FBXL12	-0.220974545	3.97414214	0.003810385	0.017225322
HECTD1	0.303988768	7.38363196	0.003931571	0.01766958
FBXL14	0.334875472	3.645838122	0.004136464	0.0183889
MARCH-11	-2.700231304	-2.289348013	0.004194816	0.018599755
TRIM68	0.415704794	4.736659963	0.004238399	0.018745102
TRAF3	0.272221372	5.124258096	0.004420992	0.019367537
NEURL3	-0.90405246	0.298623749	0.004604137	0.019951579
KLHL25	0.368685238	3.211615067	0.0050312	0.021366439
RNF43	-0.477888386	5.481953802	0.005253914	0.022015897
E4F1	0.278901507	4.662636518	0.005456436	0.022682835
TRIM16	0.474839046	4.487815554	0.005492647	0.022806316
NHLRC1	-0.430992033	1.651027536	0.005675067	0.023371489
TRIM36	-0.567793144	1.920222082	0.006937671	0.027245604
FBXO18	0.195007857	5.910099481	0.007040959	0.027562861
RNF19A	-0.383468707	6.845545301	0.007082742	0.027672888
RNF187	0.253507669	7.059542021	0.008326291	0.031215347
KCMF1	0.200229454	6.133471264	0.008584505	0.032010487
CDC16	0.200926712	5.859932847	0.008815327	0.0326622
RNF168	-0.318605472	4.525844495	0.009023971	0.03327969
DDB2	-0.298544265	4.667307329	0.009259967	0.033968618
SOCS1	-0.467391334	3.14556179	0.010223807	0.036678172
PDZRN3	-0.438301311	3.369107214	0.010306137	0.036890905
RNF220	0.183969584	6.02183125	0.010977102	0.038720179
RNF115	0.193417369	6.016376432	0.011874401	0.041043953
FBXW10	0.681116803	-2.097717831	0.012619044	0.043010923
DCAF4	0.23917912	3.976631535	0.012749258	0.043270587
WSB1	0.391856047	7.791462218	0.012899501	0.043652432
TRIM2	-0.451427753	7.162537471	0.012974806	0.043828029
FBXL7	-0.432010977	3.269632855	0.013082697	0.044100359
CRBN	-0.253192022	5.047760206	0.013833348	0.046034516

UNKL	0.259667889	3.754401336	0.014326969	0.047291715

Supplemental Table 1 logFC = logarithmic fold change, logCPM = logarithmic Counts Per Million, FDR = False Discovery Rate, DUBs = deubiquitinating enzymes, E2s = conjugating enzymes, E3s = ligase enzymes. All p values were two sides and less than 0.05 were considered significant.

Supplemental Table 2 Analysis results of 116 genes by Cox proportional hazards regression model

	LKB1 mutation		LKB1 wild type	
gene	HR (95% CI)	p	HR (95% CI)	p
DUB(16)				
COPS5	1.660(0.560-4.920)	0.361	1.421(0.864-2.339)	0.167
CYLD	0.963(0.352-2.635)	0.941	0.532(0.316-0.896)	0.018
EIF3H	1.538(0.566-4.180)	0.399	1.758(1.067-2.894)	0.027
OTUB2	1.101(0.471-2.574)	0.823	1.961(1.213-3.171)	0.006
OTUD7A	0.502(0.215-1.172)	0.111	0.573(0.351-0.934)	0.026
PAN2	0.939(0.125-7.070)	0.951	0.596(0.353-1.006)	0.053
PRPF8	0.909(0.370-2.234)	0.835	0.627(0.380-1.035)	0.627
USP2	0.036(0.000-3.990)	0.166	0.711(0.437-1.157)	0.170
USP21	-	-	1.519(0.827-2.788)	0.178
USP25	0.794(0.265-2.375)	0.680	1.482(0.924-2.378)	0.103
USP35	0.430(0.158-1.170)	0.098	0.841(0.384-1.842)	0.665
USP4	1.001(0.424-2.366)	0.998	0.489(0.303-0.790)	0.003
USP43	1.966(0.661-5.848)	0.224	1.431(0.890-2.301)	0.139
USP5	1.731(0.749-3.999)	0.199	2.421(1.495-3.922)	<0.001
USP53	0.900(0.366-2.213)	0.819	0.735(0.447-1.210)	0.226
VCPIP1	1.147(0.464-2.838)	0.766	1.683(1.007-2.813)	0.047
E2(7)				
ATG3	1.662(0.642-4.298)	0.293	1.447(0.901-2.323)	0.126
UBE2A	0.913(0.393-2.122)	0.833	2.367(1.310-4.278)	0.004
UBE2D4	1.040(0.306-3.533)	0.950	1.663(1.025-2.698)	0.039
UBE2L6	1.112(0.474-2.607)	0.807	0.708(0.432-1.160)	0.170
UBE2Q1	1.149(0.337-3.918)	0.824	1.430(0.887-2.303)	0.142
UBE2Q2L	1.473(0.539-4.024)	0.450	1.723(0.879-3.377)	0.113
UBE2U	0.252(0.034-1.884)	0.179	0.616(0.362-1.049)	0.074
E3(93)				
MARCH-1	-	-	0.418(0.242-0.719)	0.002
MARCH-3	2.331(0.935-5.810)	0.069	1.427(0.824-2.470)	0.204
MARCH-4	1.304(0.555-3.061)	0.542	1.719(1.067-2.770)	0.026
MARCH-5	0.947(0.314-2.858)	0.923	1.968(1.209-3.205)	0.006
MARCH-11	3.666(1.002-13.407)	0.050	2.001(1.092-3.666)	0.025
ANAPC16	3.955(0.530-29.502)	0.180	1.114(0.684-1.815)	0.664
AREL1	22.097(0.003-182457.296)	0.501	1.967(0.715-5.416)	0.190
ASB1	1.382(0.561-3.406)	0.482	2.126(1.151-3.927)	0.016
ASB16	0.596(0.247-1.436)	0.249	0.590(0.366-0.951)	0.030
ASB5	0.659(0.256-1.695)	0.387	1.313(0.816-2.111)	0.262

ASB9	0.581(0.208-1.620)	0.299	1.354(0.844-2.172)	0.210
BIRC7	1.332(0.554-3.204)	0.522	0.545(0.338-0.877)	0.012
BTBD6	0.759(0.289-1.996)	0.576	1.405(0.870-2.268)	0.164
CAP1	6.870(1.478-31.931)	0.014	1.358(0.790-2.332)	0.268
CDC16	1.886(0.252-14.105)	0.537	0.697(0.402-1.209)	0.199
CDH1	0.890(0.328-2.419)	0.819	0.936(0.580-1.510)	0.785
CMYA5	0.965(0.376-2.477)	0.942	0.720(0.435-1.190)	0.200
CRBN	0.480(0.204-1.129)	0.093	0.540(0.314-0.929)	0.026
DCAF4	0.320(0.133-0.769)	0.011	0.875(0.540-1.416)	0.586
DCAF8	2.758(0.640-11.880)	0.173	0.713(0.444-1.144)	0.161
DDB2	1.412(0.580-3.439)	0.447	1.290(0.798-2.086)	0.298
E4F1	20.964(0.000-1035806875)	0.736	21.855(0.116-4125.364)	0.249
FBXL12	0.533(0.195-1.456)	0.220	0.781(0.485-1.259)	0.311
FBXL13	1.394(0.581-3.345)	0.456	1.584(0.983-2.554)	0.059
FBXL14	0.783(0.336-1.828)	0.572	2.163(1.340-3.493)	0.002
FBXL6	1.252(0.460-3.408)	0.661	1.488(0.921-2.405)	0.104
FBXL7	0.688(0.203-2.333)	0.548	0.505(0.283-0.903)	0.021
FBXL8	1.054(0.411-2.704)	0.913	0.567(0.339-0.947)	0.030
FBXO18	0.560(0.240-1.308)	0.181	0.714(0.436-1.169)	0.181
FBXO32	1.706(0.725-4.010)	0.221	2.258(1.334-3.824)	0.002
FBXO40	0.528(0.205-1.361)	0.186	0.980(0.609-1.578)	0.934
FBXO46	1.354(0.527-3.476)	0.529	1.966(1.196-3.231)	0.008
FBXW10	1.917(0.702-5.235)	0.204	1.618(0.771-3.394)	0.203
FBXW5	1.111(0.371-3.326)	0.851	1.554(0.964-2.507)	0.071
HECTD1	1.042(0.307-3.544)	0.947	0.716(0.445-1.152)	0.169
HERC5	1.047(0.431-2.546)	0.919	0.664(0.396-1.112)	0.120
HERC6	1.081(0.249-4.685)	0.917	0.783(0.474-1.295)	0.341
IRF2BP1	0.622(0.259-1.492)	0.287	2.730(1.571-4.746)	<0.001
KBTBD8	0.552(0.231-1.319)	0.181	0.693(0.410-1.171)	0.171
KCMF1	1.941(0.753-5.000)	0.170	2.265(1.399-3.666)	0.001
KCTD10	1.669(0.684-4.070)	0.260	0.593(0.365-0.963)	0.035
KLHL25	0.802(0.106-6.081)	0.831	1.265(0.702-2.281)	0.434
KLHL7	1.878(0.782-4.508)	0.158	1.088(0.651-1.816)	0.748
LRSAM1	0.733(0.309-1.740)	0.482	1.063(0.646-1.750)	0.809
LTN1	0.979(0.404-2.372)	0.963	0.679(0.420-1.099)	0.115
MAGEC2	2.494(0.317-19.602)	0.385	1.586(0.911-2.761)	0.103
MID2	0.413(0.152-1.127)	0.084	0.736(0.457-1.186)	0.208
MUL1	1.000(0.000-383075.678)	1.000	2.640(1.512-4.610)	0.001
NEDD4L	0.493(0.213-1.142)	0.099	0.762(0.471-1.234)	0.269
NEURL3	0.639(0.259-1.573)	0.329	1.094(0.646-1.853)	0.737

NHLRC1	0.044(0.000-52.996)	0.387	1.066(0.651-1.746)	0.800
PDZRN3	0.483(0.209-1.117)	0.089	0.738(0.442-1.230)	0.244
PML	2.826(1.160-6.884)	0.022	1.172(0.727-1.889)	0.515
PPIL2	1.307(0.477-3.579)	0.603	2.187(1.252-3.820)	0.006
PRKN	0.374(0.162-0.866)	0.022	0.785(0.479-1.286)	0.337
RAB40AL	0.614(0.250-1.508)	0.287	0.737(0.458-1.185)	0.207
RAB40B	0.133(0.046-0.386)	<0.001	0.658(0.379-1.140)	0.135
RAB40C	0.490(0.158-1.517)	0.216	1.081(0.658-1.778)	0.758
REN	0.046(0.000-319.220)	0.494	0.816(0.500-1.332)	0.416
RNF115	0.988(0.428-2.284)	0.978	0.746(0.435-1.281)	0.288
RNF144B	1.120(0.474-2.646)	0.796	0.579(0.359-0.936)	0.026
RNF167	1.093(0.444-2.690)	0.846	1.324(0.824-2.126)	0.246
RNF168	4.234(1.176-15.250)	0.027	1.713(0.945-3.104)	0.076
RNF187	0.408(0.169-0.986)	0.408	1.573(0.962-2.572)	0.071
RNF19A	0.970(0.412-2.284)	0.945	0.527(0.325-0.855)	0.009
RNF220	2.123(0.915-4.928)	0.080	1.992(1.188-3.341)	0.009
RNF43	0.550(0.226-1.341)	0.189	0.700(0.394-1.243)	0.223
SH3RF1	0.356(0.136-0.929)	0.035	0.902(0.588-1.461)	0.676
SH3RF2	2.771(0.593-12.951)	0.195	1.586(0.969-2.595)	0.066
SMURF2	0.578(0.248-1.347)	0.204	0.717(0.421-1.219)	0.219
SOCS1	0.510(0.207-1.257)	0.143	0.481(0.285-0.810)	0.006
SOCS7	0.601(0.255-1.416)	0.244	0.775(0.475-1.264)	0.307
SPSB4	0.435(0.169-1.117)	0.084	0.589(0.353-0.981)	0.042
TRAF2	0.538(0.155-1.866)	0.329	1.189(0.735-1.924)	0.481
TRAF3	3.485(1.394-8.709)	0.008	1.134(0.561-2.293)	0.727
TRIM14	1.340(0.573-3.135)	0.499	0.824(0.511-1.328)	0.426
TRIM16	2.135(0.909-5.013)	0.081	2.015(1.241-3.269)	0.005
TRIM16L	1.704(0.735-3.952)	0.214	1.509(0.888-2.565)	0.128
TRIM17	0.473(0.192-1.164)	0.103	1.070(0.650-1.761)	0.790
TRIM2	0.253(0.103-0.620)	0.003	0.675(0.388-1.175)	0.165
TRIM22	0.545(0.160-1.853)	0.331	0.585(0.363-0.943)	0.028
TRIM34	1.408(0.475-4.177)	0.537	0.514(0.289-0.914)	0.023
TRIM36	0.980(0.288-3.337)	0.974	1.407(0.847-2.339)	0.187
TRIM37	1.367(0.571-3.269)	0.483	2.892(1.428-5.858)	0.003
TRIM46	0.582(0.250-1.354)	0.209	0.898(0.558-1.446)	0.658
TRIM50	0.219(0.080-0.602)	0.003	0.568(0.352-0.918)	0.021
TRIM53AP	1.058(0.308-3.632)	0.928	0.927(0.506-1.698)	0.806
TRIM55	0.207(0.028-1.541)	0.124	0.527(0.321-0.864)	0.011
TRIM68	0.180(0.062-0.520)	0.002	0.516(0.300-0.887)	0.017
TRIM72	0.568(0.167-1.929)	0.364	0.820(0.439-1.534)	0.535

TRIML2	3.130(1.280-7.653)	0.012	1.187(0.668-2.109)	0.559
UNKL	0.984(0.130-7.442)	0.988	0.490(0.281-0.855)	0.012
WSB1	0.586(0.252-1.361)	0.214	0.607(0.373-0.988)	0.045

Supplemental Table 2 Analysis results of 116 genes by Cox proportional hazards regression model. All p values were two sides and less than 0.05 were considered significant. HR= hazard ratio, CI= confidence interval.

Supplemental Table 3 Primers for Real-time PCR

Primers	Forward	Reverse
STK11	TGATGGAGTACTGCGTGTGT	GCTTGATGTCCTTGTGCACA
SH3RF1	TGGGACCAACTGCACAAATG	GGTCTCCAGGCTCTTTTCCT
TRAF3	AGGCGTGTAAATACCGGGAA	GCCTTGATCTGCTGGTTTGT
TRIM2	TGGAGAAGGAAATGGGCAGT	CTGCAACCACAACATGACCA
18S rRNA	AAACGGCTACCACATCCAAG	CCTCCAATGGATCCTCGTTA
